# Bilateral diffuse xanthogranulomatous pyelonephritis in end-stage renal disease (ESRD)

**DOI:** 10.1590/2175-8239-JBN-2020-0242

**Published:** 2021-05-28

**Authors:** Milena Regina dos Santos Perez, Mirele Cristine Santos de Oliveira, Danielle Bispo Vieira Ortiz, Juliana Abeche Fermozelli, William Luis Oliveira, Ronaldo D'Avila

**Affiliations:** 1 Pontifícia Universidade Católica de São Paulo, Faculdade de Ciências Médicas e da Saúde, Sorocaba, SP, Brasil.; 2 Universidade de Marília, Marília, SP, Brasil.; 3 Pontifícia Universidade Católica de São Paulo, Faculdade de Ciências Médicas e da Saúde, Departamento de Cirurgia, Sorocaba, SP, Brasil.; 4 Conjunto Hospitalar de Sorocaba, Sorocaba,SP, Brasil.

Xanthogranulomatous pyelonephritis (XGP) is a very rare form of kidney disease characterized by destruction of renal parenchyma, its fibrosis, and its replacement by lipid-laden macrophages. 1,2.

**Figure 1:**
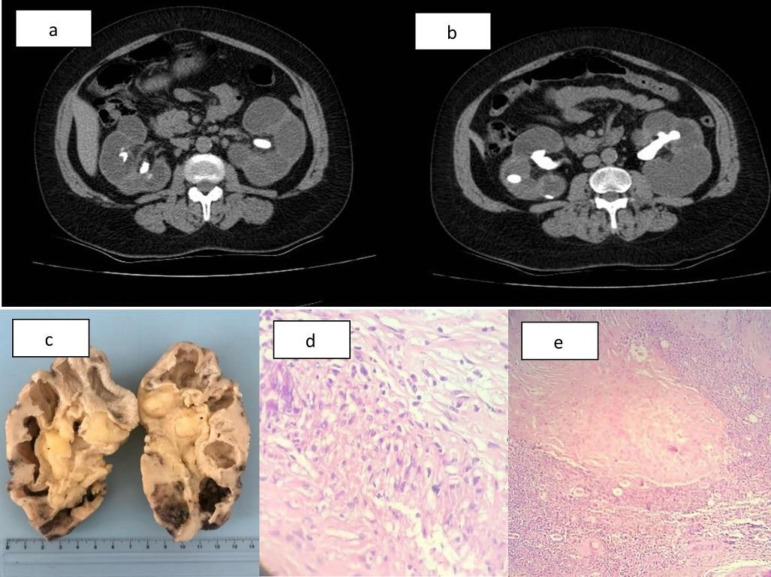
CT scan showing the “bear paw sign” in both kidneys (a) and staghorn stones with bilateral hydronephrosis (b). Gross pathological examination showing bilateral cystic formations, abscesses, and fibrosis with architectural distortion of the renal parenchyma (c). Optical microscopy showing numerous macrophages, some with xanthomized aspect (d) and renal parenchyma with focus of histiocytic reaction and areas of necrosis (e).

We describe a case of a 46-year-old woman with bilateral staghorn stones and end-stage renal disease (ESRD) under renal replacement therapy by hemodialysis. An abdominal CT scan suggested bilateral XGP (Figure 1) and she was submitted to bilateral nephrectomy. Histopathological analysis confirmed the diagnosis.

Complications in XGP can occur, such as cortical atrophy, abscesses, and kidney loss. The treatment of choice is total nephrectomy, and tumors and granulomatous diseases should be included in the differential diagnosis^3^-^5^.
